# Energy Conservation in Fermentations of Anaerobic Bacteria

**DOI:** 10.3389/fmicb.2021.703525

**Published:** 2021-09-13

**Authors:** Wolfgang Buckel

**Affiliations:** Laboratorium für Mikrobiologie, Fachbereich Biologie, Philipps-Universität Marburg, Marburg, Germany

**Keywords:** ΔμNa^+^, decarboxylation, ferredoxin, Rnf, electron bifurcation, coenzyme B_12_, glycyl radical enzymes, oxygen sensitivity

## Abstract

Anaerobic bacteria ferment carbohydrates and amino acids to obtain energy for growth. Due to the absence of oxygen and other inorganic electron acceptors, the substrate of a fermentation has to serve as electron donor as well as acceptor, which results in low free energies as compared to that of aerobic oxidations. Until about 10 years ago, anaerobes were thought to exclusively use substrate level phosphorylation (SLP), by which only part of the available energy could be conserved. Therefore, anaerobes were regarded as unproductive and inefficient energy conservers. The discovery of electrochemical Na^+^ gradients generated by biotin-dependent decarboxylations or by reduction of NAD^+^ with ferredoxin changed this view. Reduced ferredoxin is provided by oxidative decarboxylation of 2-oxoacids and the recently discovered flavin based electron bifurcation (FBEB). In this review, the two different fermentation pathways of glutamate to ammonia, CO_2_, acetate, butyrate and H_2_ via 3-methylaspartate or via 2-hydroxyglutarate by members of the *Firmicutes* are discussed as prototypical examples in which all processes characteristic for fermentations occur. Though the fermentations proceed on two entirely different pathways, the maximum theoretical amount of ATP is conserved in each pathway. The occurrence of the 3-methylaspartate pathway in clostridia from soil and the 2-hydroxyglutarate pathway in the human microbiome of the large intestine is traced back to the oxygen-sensitivity of the radical enzymes. The coenzyme B_12_-dependent glutamate mutase in the 3-methylaspartate pathway tolerates oxygen, whereas 2-hydroxyglutaryl-CoA dehydratase is extremely oxygen-sensitive and can only survive in the gut, where the combustion of butyrate produced by the microbiome consumes the oxygen and provides a strict anaerobic environment. Examples of coenzyme B_12_-dependent eliminases are given, which in the gut are replaced by simpler extremely oxygen sensitive glycyl radical enzymes.

## Introduction

Fermentation is a chemical reaction catalyzed by living cells with two different meanings. In biotechnology the bacterial production of a certain compound mainly from glucose under aerobic conditions is called fermentation, e.g., L-lysine fermentation. In this review, however, fermentation is defined as an anaerobic bacterial redox process of an organic substrate leading to different products, e.g., fermentation of glutamate to ammonia, CO_2_, acetate, butyrate and hydrogen. Thereby glutamate is oxidized to CO_2_ and acetate; the resulting reducing equivalents are used to synthesize butyrate and H_2_ to establish a balanced chemical equation. Inorganic electron acceptors such as nitrate, sulfate or Fe(III) are not involved; otherwise the process would be called respiration rather than fermentation. Thermodynamically, a fermentation is an exergonic process with a free energy of ΔG°′ <−20 kJ/mol. ΔG° is defined for 1 M substrates and products, 10^5^ Pa for gasses and at a temperature of 298 K. Under biological conditions, in dilute aqueous solutions (55.5 M H_2_O) and at pH 7.0, ΔG°′ is used. In this review, the ΔG°′ values have been calculated from the data given by Thauer et al. (1977).

A well-established fermentation is the conversion of glucose by lactic acid bacteria to lactate at pH 7.0 with ΔG°′ = −185 kJ/mol (eq. 1). In contrast, the aerobic oxidation of glucose in mammalian cells with O_2_ to CO_2_ and H_2_O yields ΔG°′ = −2,872 kJ/mol (eq. 2).


(1) CH6O12→62CH-3CHOH-COO+-2H;+ ΔG°=′-185kJ/mol

(2) CH6O12+66O→26CO+26HO2; ΔG°=′-2,872kJ/mol

Glucose oxidation via glycolysis, pyruvate dehydrogenase, Krebs’ (citric acid) cycle and mitochondrial respiratory chain are very well analyzed processes leading to about 38 mol ATP/mol glucose; (−2,872 kJ/mol glucose): (38 mol ATP/mol glucose) = −76 kJ/mol ATP. Hence in a catabolic reaction with one or more irreversible steps, a free energy change of −76 kJ is required to generate 1 mol ATP, whereas under equilibrium conditions only about −50 kJ are necessary (Thauer et al., 1977; Buckel and Thauer, 2013). If this value is applied to the fermentation of glucose to 2 lactate, the ATP yield should be (−185 kJ/mol): (−76 kJ/mol ATP) = 2.4 ATP, which is 20% higher than 2.0 ATP via substrate level phosphorylation in glycolysis. This examples tells why energy conservation in fermentative anaerobic bacteria has been considered as inefficient. The discovery that anaerobic bacteria are able use in addition electrochemical ion gradients for ATP synthesis, catalyzed by ubiquitous H^+^ or Na^+^ dependent F_1_F_0_-ATP-synthases (von Ballmoos et al., 2009), changed this view. Work by Konings and colleagues showed that in the glucose fermenting *Streptococcus cremoris*, the export of a formed lactic acid molecule is accompanied by a proton, which establishes an electrochemical proton gradient (Otto et al., 1980). Since 2 lactic acids are produced from glucose, two additional protons are exported which can give rise to 0.5 ATP catalyzed by ATP synthase. Thus the involvement of electrochemical ion gradients allows bacteria to use energy increments that are smaller than that of 1 ATP, which makes anaerobes even more efficient than aerobes. Therefore, the amount of energy to synthesize 1 ATP in anaerobes is now considered as about −66 kJ rather than −76 kJ in aerobes; see section “Fermentation of Glutamate: 2-Hydroxyglutarate or 3-Methylaspartate Pathway?”

In this review, I will show that also anaerobes have enzymes which are able to generate a Na^+^/H^+^ motive force, the biotin-containing decarboxylases (see section “Biotin-Containing Decarboxylases as Sodium Ion Pumps”) and the NAD:ferredoxin oxidoreductase (Rnf, see section “The Na+/H+ Pump Ferredoxin:NAD Oxidoreductase Also Called Rnf”). Sections “Flavin Based Electron Bifurcation” and “On the Mechanism of Electron Bifurcation Catalyzed by EtfAB-Bcd” will give an introduction to flavin based electron bifurcation, by which “energy rich” reduced ferredoxin and flavodoxin are obtained. In section “Fermentation of Glutamate: 2-Hydroxyglutarate or 3-Methylaspartate Pathway?” the 2-hydroxyglutarate and the 3-methylaspartate pathway of the fermentation of glutamate are introduced, which apply the biotin-containing decarboxylases and Rnf together with substrate level phosphorylation (SLP) to obtain the maximum theoretical yield of ATP. Section “Oxygen-Tolerant and -Intolerant Radical Enzymes” tackles the question why nature evolved two different pathways of glutamate fermentation. Finally, section “Butyrate Provides Anaerobiosis in the Gut” shows how butyrate converts the human gut to an extremely oxygen-free environment and an ideal environment for strict anaerobic bacteria.

## Biotin-Containing Decarboxylases as Sodium Ion Pumps

Concomitant with the discovery of Konings, bacterial Na^+^ pumps were detected and isolated, driven by decarboxylation (Buckel, 2001a). The first decarboxylase of this type was detected by Dimroth (1980) in *Klebsiella pneumoniae* and characterized in the following years as biotin containing integral membrane enzyme catalyzing the decarboxylation of oxaloacetate to pyruvate, ΔG°′ ≈ −30 kJ/mol coupled to the translocation of 2 Na^+^. This breakthrough was followed by methylmalonyl-CoA decarboxylase from *Veillonella aerogenes* (Hilpert and Dimroth, 1982) and glutaconyl-CoA decarboxylases from *Acidaminococcus fermentans, Peptostreptococcus asaccharolyticus* (formerly called *Peptococcus aerogenes;* Ezaki et al., 1983) and *Clostridium symbiosum* (Buckel and Semmler, 1982, 1983). These enzymes share a common two step mechanism: the carboxyl group of the substrate is transferred to enzyme bound biotin. Addition of a proton to the carbonyl group of the formed N-carboxybiotin causes decarboxylation, which drives the translocation of Na^+^ ions from the cytoplasm through the membrane to the outside of the bacterium. These biotin containing decarboxylases are composed of 3–5 subunits. The largest and hydrophilic α-subunit catalyzes the carboxy transfer from substrate to biotin and the extremely hydrophobic β-subunit is responsible for the decarboxylation of carboxybiotin and Na^+^ transport. Biotin is covalently attached to a conserved lysine residue of the γ-subunit and the small δ-subunit connects the α-subunit in the cytoplasm with the β-subunit in the membrane (Vitt et al., 2020). Variations are observed with the γ-subunit. In oxaloacetate decarboxylase from *K. aerogenes*, a small domain of the α-subunit serves as γ-subunit (Schwarz et al., 1988; Xu et al., 2020), whereas glutaconyl-CoA decarboxylase from *C. symbiosum* contains two slightly different γ-subunits (Kress et al., 2009). The amount of Na^+^ ions transported by these pumps could be determined for oxaloacetate decarboxylase from *K. pneumoniae* as up to 2 Na^+^ per decarboxylation (Dimroth and Thomer, 1993). This is supported by the recent cryo-EM structure (4.5 Å resolution) of the trimeric βγ-subunit complex of oxaloacetate decarboxylase from *Salmonella typhimurium*, where two Na^+^ binding sites in one β-subunit were detected. Unfortunately, the coupling between decarboxylation of carboxybiotin and Na^+^-transport could not be elucidated (Xu et al., 2020).

Oxaloacetate decarboxylase from *K. pneumoniae* is involved in the fermentation of citrate to 2 acetates, 1.2 CO_2_ and 0.5 formate. The pathway involves citrate cleavage to acetate and oxaloacetate, decarboxylation of the latter to CO_2_, pyruvate and ΔμNa^+^, cleavage of pyruvate with CoA to acetyl-CoA and formate, substrate level phosphorylation (SLP) with acetyl-CoA to acetate, CoA and ATP. Generation of ΔμNa^+^ is used for citrate transport and for driving the endergonic reduction of NAD^+^ for anabolic purposes by formate via ubiquinol (Pfenninger-Li and Dimroth, 1992). A balanced stoichiometry of citrate fermentation would give ΔG°′ = −78.7 kJ/mol, which is the amount of energy required for the synthesis of about 1.2 ATP (eq. 3).


(3) Citrate+3-HO2→CO+2formate+-2acetate;-ΔG′°=-78.7kJ/mol

*Propionigenium modestum* thrives from an apparently very simple chemical reaction, the decarboxylation of succinate to propionate (eq. 4) (Hilpert et al., 1984). Propionate CoA-transferase converts succinate to succinyl-CoA, which is rearranged to (*R*)-methylmalonyl-CoA in a coenzyme B_12_-dependent manner. Epimerization affords (*S*)-methylmalonyl-CoA, which is decarboxylated to propionyl-CoA, whereby ΔμNa^+^ is generated (Hilpert and Dimroth, 1982). At the low succinate concentrations in the Canale Grande in Venice, Italy (ca. 1 mM), from which the organism was isolated (Schink, 1982), the free energy is sufficient for about ½ ATP (eq. 4). ΔμNa^+^ directly drives the Na^+^-dependent F_1_F_*o*_-ATP synthase (Laubinger and Dimroth, 1988).


(4) Succinate+2-H→+propionate+-CO;2ΔG°=′-24.4kJ/mol;at1mMconcentrationsΔG*=′-43.5kJ/mol

## The Na^+^/H^+^ Pump Ferredoxin:Nad Oxidoreductase Also Called Rnf

**FIGURE 1:**
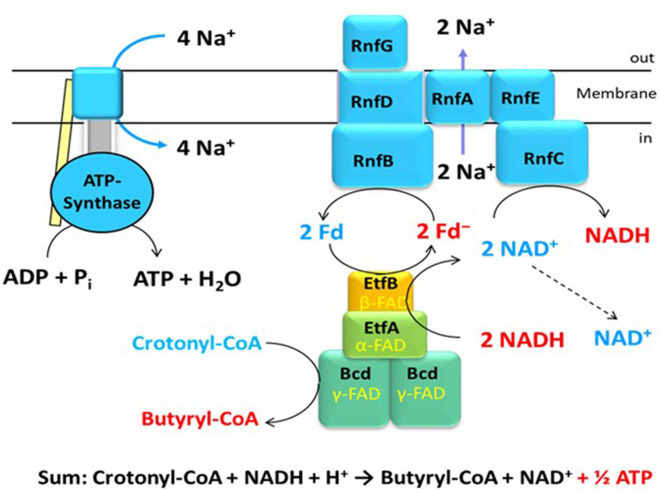
An example of energy conservation in butyrate producing anaerobic bacteria via the generation of an ion motif force. The bifurcating EtfAB-Bcd complex reduces 2 Fd with NADH driven by the exergonic reduction of crotonyl-CoA to butyryl-CoA with a second NADH. The Rnf complex in the membrane produces 2 ΔμNa^+^ from the exergonic reduction of NAD^+^ by 2 Fd^–^. The ATPase generates 1 ATP from 4 ΔμNa^+^.

The genes coding for Rnf have been known from *Rhodobacter capsulatus*, where it is involved in nitrogen fixation and named Rnf (for *Rhodobacter* nitrogen fixation) (Schmehl et al., 1993). Similar genes were detected in the genome of *C. tetani*, a close relative of *C. tetanomorphum*, whose membranes catalyzed the oxidation of NADH with ferricyanide [hexacyanoferrate(III)] (Brüggemann et al., 2003; Imkamp et al., 2007). The enzyme complex in membranes from *C. tetanomorphum*, which contained the highest activity, could be solubilized with dodecylmaltoside and purified (Boiangiu et al., 2005; Jayamani, 2008). SDS-PAGE indicated that the complex was composed of six different subunits RnfCDGEAB (Figure 1), RnfCDGE are related to four subunits of the sodium pumping NADH:quinone oxidoreductase (Nqr) from *Vibrio cholerae* (Steuber et al., 2014a,b). The yellow-brownish native enzyme complex contained covalently bound flavin, non-covalently bound FMN, riboflavin and 23 ± 1 mol Fe per mol protein, most likely in six iron-sulfur clusters (Boiangiu et al., 2005).

The oxidation of NADH with ferricyanide catalyzed by Rnf in membrane vesicles from *C. tetanomorphum* showed no Na^+^-dependence; probably the electrons did not pass through all six subunits due to a short cut. Therefore, the reaction was performed in reverse. The reduction of NAD^+^ was measured in an assay, in which 1 mM Ti(III)citrate was used to keep ferredoxin in the reduced state. Controls showed that Ti(III)citrate reduced NAD^+^ without ferredoxin and membranes at pH ≥ 7.0. But at pH 6.8 again no Na^+^-dependence could be observed (Imkamp et al., 2007; Jayamani, 2008). The first Na^+^-transport with an Rnf was demonstrated with membrane vesicles from *Acetobacterium woodii*, NAD^+^, Ti(III)citrate and ^22^Na^+^ by Biegel and Müller (2010). The electrogenic transport was stimulated by valinomycin in the presence of K^+^ and inhibited by 100 μM Na^+^-ionophore N,N,N’,N’-tetracyclohexyl-1,2-phenylendioxydiacetamide (ETH 2120). Later Ti(III)citrate was replaced by purified carbon monoxide dehydrogenase/acetyl-CoA synthase and CO as reductant for ferredoxin, which resulted in much more reliable dependencies on Na^+^ (Hess et al., 2013). In a similar manner reduced ferredoxin and flavodoxin quinol were generated with the electron bifurcating electron transfer flavoprotein (EtfAB) and butyryl-CoA dehydrogenase (Bcd) both from *A. fermentans* (see also next chapter, eq. 5). With membranes from *A. fermentans* it was shown that Rnf activity was Na^+^-dependent with apparent *K*_*m*_ = 120 ± 20 μM Na^+^ and *K*_*m*_ = 280 ± 50 μM Li^+^ at pH 6.8; in addition, *K*_*m*_ = 2.0 ± 0.4 μM flavodoxin and 1.4 ± 0.1 μM ferredoxin were measured (Chowdhury et al., 2016). The values for Rnf from *A. woodii* were: *K*_*m*_ = 201 ± 30 μM Na^+^ at pH 6 and *K*_*m*_ = 155 ± 39 μM Na^+^ at pH 7.7 (Hess et al., 2013). Surprisingly, the *K*_*m*_ values for Na^+^ and Li^+^ are much lower than those of the biotin-containing Na^+^ pumps glutaconyl-CoA decarboxylase from A. *fermentans* (Buckel and Semmler, 1982) or oxaloacetate decarboxylase from *K. pneumoniae* (Dimroth and Thomer, 1986), which exhibit apparent *K*_*m*_ values of 1.0–1.5 mM Na^+^ and 25–100 mM Li^+^. Recently a survey showed that not all anaerobic bacteria contain Rnfs and not all Rnfs are Na^+^-dependent. Thus the Rnf activity is absent in *R. capsulatus* and *Escherichia coli*, but present also in *Bacteroides fragilis*, *Clostridium ljungdahlii*, *V. cholerae*, and *Clostridium kluyveri.* Besides *C. tetanomorphum*, *A. fermentans*, and *A. woodii*, a Na^+^-dependence could be only detected in Rnf from *B. fragilis* (Hess et al., 2016).

## Flavin Based Electron Bifurcation

Ferredoxin-dependent electron bifurcation represents an important additional process to generate the energy rich molecule. Only a few enzymes, such as pyruvate ferredoxin: oxidoreductases (PFOR), reduce ferredoxin, the substrate of Rnf. A search for a reductant other than PFOR, took the butyryl-CoA dehydrogenases (Bcd) into consideration, because the difference between the reduction potentials of NAD/NADH, *E*_0_’ = −320 mV, and crotonyl-CoA/butyryl-CoA, *E*_0_’ = −10 mV, amounts to +310 mV, equal to ΔG°′ = −*n* × *F* × Δ*E*_*o*_’ = −2 × 96.5 × 0.310 Volt = −59.8 kJ mol^–1^. Probably Bcd also reduces ferredoxin or pumps Na^+^ driven by this large ΔG°’. The hypothesis of a membrane bound butyryl-CoA dehydrogenase (Bcd) could be refuted by the already known isolation of a soluble green homotetrameric Bcd from *A. fermentans* which could be separated from the yellow heterodimeric electron transferring flavoprotein (EtfAB) (Buckel, 1990). A very similar green soluble Bcd has been known from *Megasphaera elsdenii* (formerly called *Peptostreptococcus elsdenii*) (Engel and Massey, 1971), which like *A. fermentans* belongs to the *Negativicutes*, a class with a Gram-negative cell wall of the, predominantly, Gram-positive phylum *Firmicutes* (Latin: cutis firma = strong outer skin) (Marchandin et al., 2010). In contrast, butyryl-CoA dehydrogenase from the Gram-positive *C. tetanomorphum* forms a tight complex with the heterodimeric EtfAB (EtfAB-Bcd). But immunogold labeling and electron microscopy clearly showed that this EtfAB-Bcd complex was also not associated with the membrane (Herrmann, 2008). Nevertheless, a hypothesis arose from these negative results that the reduction of crotonyl-CoA with NADH catalyzed by Bcd from *C. tetanomorphum* was somehow involved in the reduction of ferredoxin (*E*_*o*_’ = −420 mV). Furthermore, since 1969 it was known that cell extracts of *Clostridium kluyveri* catalyzed an acetyl-CoA dependent reduction of ferredoxin with NADH (Thauer et al., 1969). Since the cell extract contained all enzymes for the synthesis of butyrate (Seedorf et al., 2008) as did *C. tetanomorphum* cells, it was ferredoxin that indeed should be reduced by NADH. Similar to complex III of the mitochondrial respiratory chain, it was postulated that NADH reduced EtfAB, which bifurcated one electron exergonically to δ-FAD of Bcd and the other endergonically to ferredoxin (eq. 5) (Herrmann et al., 2008). To reduce crotonyl-CoA with two electrons, repetition of the reaction is required:


(5) 2NADH+Crotonyl-CoA+2Fd→2NAD++Butyryl-CoA+2Fd;-ΔE=o′+210mV;ΔG°=′-40.5kJ/mol

This hypothesis was verified first with the purified Etf-Bcd complex from *C. kluyveri* (Li et al., 2008). The catalytic amounts of purified ferredoxin from *Clostridium pasteurianum* were regenerated with purified [FeFe]hydrogenase also from *C. pasteurianum*. If one of the components of this assay (NADH, crotonyl-CoA, Fd, hydrogenase or EtfAB-Bcd) was omitted, no reaction could be observed. Hence the electron bifurcation system is tightly coupled. The purified Etf-Bcd complex from *C. tetanomorphum* catalyzed exactly the same reaction as in eq. 5, as did the combination of Bcd and EtfAB from *A. fermentans* or *M. elsdenii* (Chowdhury et al., 2014, 2015).

## On the Mechanism of Electron Bifurcation Catalyzed by EtfAb-Bcd

**FIGURE 2:**
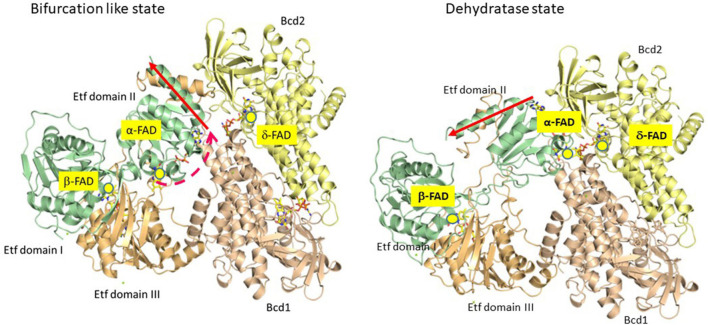
Partial crystal structures of the recombinant Etf-Bcd complex from *C. difficile* produced in *E. coli;* EtfAB and 2 subunits of the tetrameric Bcd are displayed. The dehydratase state shows the structure as solved. In the bifurcation state domain II of Etf rotated CW by 80° as found in Etf from *A. fermentans* (see red arrow)*;* Etf, domains I + II (subunit A) in green, domain III (subunit B) in light brown, Bcd1 in yellow and Bcd2 in pinkish-brown. In the dehydratase state, α-FAD and δ-FAD are located close together (8 Å distance), ready for ET. In the bifurcation-like state, α-FAD and β-FAD are 18 Å apart; further rotation by 10° CW would bring them closer together by 4 Å, ready for electron bifurcation (taken from Buckel and Thauer, 2018a).

To study the mechanism of electron bifurcation, EtfAB-Bcd was chosen, because it contains no iron-sulfur cluster and in the resting state, it is state stable under air; for other bifurcating systems see: Buckel and Thauer (2018a, b) and Kayastha et al. (2021). EtfAB-Bcd harbors three differently bound FAD molecules, α- and β-FAD in EtfAB, and δ-FAD, one in each subunit of the tetrameric Bcd (Figure 1). As source for EtfABs, *A. fermentans* (Chowdhury et al., 2014) and *M. elsdenii* (Chowdhury et al., 2015; Vigil et al., 2021) are the most suitable organisms, because their EtfABs form no tight complexes with their Bcds and can be studied separately. The crystal structure EtfAB from *A. fermentans* (*Af*-EtfAB) revealed its composition of 3 domains, similar to those of Etfs involved in fatty acid and anaerobic toluene oxidations (Figure 2, Bifurcation-like state) (Chowdhury et al., 2014; Vogt et al., 2019). Domains I and II form subunit A and domain III subunit B. α-FAD is located in domain II (subunit A) with its isoalloxazine ring at the interface to domain III (subunit B). β-FAD sits on domain III with its isoalloxazine ring at the interface between domains III and I. β-FAD is only present in bifurcating Etfs, whereas in non-bifurcating Etfs, e.g., those acting as electron acceptors of acyl-CoA dehydrogenases, AMP alone occupies the place of the AMP part of β-FAD; the place for the isoalloxazine part is empty (Roberts et al., 1996). In *Af*-EtfAB, the distance between α- and β-FAD is 18 Å; the possible rotation of α-FAD on the flexible domain II toward β-FAD by about 10° shortens the distance between the two FADs to 14 Å, the longest distance of fast electron tunneling (Moser et al., 1992). NADH is located close to β-FAD. Since the already known structure of Bcd from *M. elsdenii* did not tell anything about the interaction with Etf (Djordjevic et al., 1995), the structure of the tight EtfAB-Bcd complex from *Clostridium difficile* was solved (Figure 2, Dehydrogenase state) (Demmer et al., 2017). The structure revealed the homotetrameric Bcd with one EtfAB at each subunit, (EtfAB)_4_(Bcd)_4_. Furthermore, α-FAD on the flexible domain II has rotated by about 80° in the opposite direction toward δ-FAD of Bcd, until 8 Å distance. Thus the two structures represent two states of the bifurcating system. The structure of EtfAB alone is close to the bifurcating state with a distance between α-FAD and β-FAD of 18 Å. In complex with Bcd, α-FAD swings into the dehydratase D-state, which brings it 34 Å apart from β-FAD (Figure 2).

**FIGURE 3:**
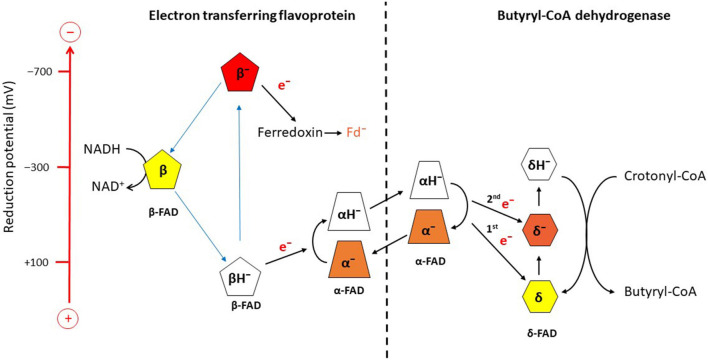
Scheme of electron bifurcation in the EtfAB-Bcd complex. The different FADs are placed according to their approximate reduction potential. NADH reduces β-FAD to β-FADH^–^, which bifurcates. One electron goes to α-FAD^–^ and the formed semiquinone, β-FAD^•−^, reduces ferredoxin (Fd) to Fd^–^. The formed hydroquinone (α-FADH^–^) swings over to Bcd and transfers one electron to δ-FAD. Note the decrase of the reduction potential of α-FAD^•−^ due to the change in location from Etf to Bcd. Repetition of this process yields δ-FADH^–^, which reduces crotonyl-CoA to butyryl-CoA. The FAD semiquinones are red, the quinones yellow and the hydroquinones colorless (Sucharitakul et al., 2021a,b).


Potentiometric titrations with dithionite under anaerobic conditions followed by UV/vis spectroscopy revealed the reduction potentials *E*_0_’ for EtfAB, α-FAD/α-FAD^•–^ = +134 mV, α-FAD^•–^/α-FADH^–^ = −36 mV, β-FAD/β-FADH^•–^ = −271 mV; for Bcd, δ-FAD/δ-FAD^•–^ = −42 mV, δ-FAD^–^/δ-FADH^–^ = −64 mV (Figure 3; Sucharitakul et al., 2021b). The one-electron reduction potentials of β-FAD could not be measured by the applied method, because the half-life of the semiquinone is expected to be extremely short, T_1/2_ ca. 10 ps (Lubner et al., 2017). In analogy to the long-known Q-cycle in the mitochondrial complex III, a reactive β-FAD^•–^ with an extremely low stability constant (log *K*_*s*_<<0) is necessary for electron bifurcation (eq. 6) (Bergdoll et al., 2016). Therefore, the β-FAD/β-FAD^•–^ reduction potential must be much more negative than that of β-FAD^•–^/β-FADH^–^, whereas in flavoproteins with stable semiquinones the opposite is the case (log *K*_*s*_ > 0), e.g., flavodoxin from *A. fermentans* with reduction potentials for FAD/FAD^–^ = −60 mV are much higher than that for FAD^–^/FADH^–^ = −420 mV (Hans et al., 2002).

(6) Ks=[FAD•-]2[FAD]×[FADH]-≤10≥-1410-21

To convert *K*_*s*_ to the one-electron reduction potentials of the bifurcating cofactor with a reduction potential of *E*_0_’, the relationship of eq. 7 is used. A graphical plot of *E*’ versus log *K*_*s*_ gives two straight lines which cross at *E*’ = *E*_*o*_’ and log *K*_*s*_ = 0. At log *K*_*s*_ > 0, the potentials are normal and at log *K*_*s*_ < 0 the potentials are inverse or called “crossed” (Nitschke and Russell, 2012).


(7) E=′±(2.3RT1/2F×-1logK)s+E′o

E=′±(0.0295×logK)s+E;o′atT=298K

Two values of *K*_*s*_ were determined experimentally, 10^–14^ to 10^–15^ with the cytochrome *bc*_1_ complex III (Zhang et al., 2007) and 10^–21^ with the bifurcating archaeal enzyme NAD^+^ ferredoxin-NADP reductase (Nfn), which reversibly bifurcates the electrons from NADPH to ferredoxin and NAD^+^ (eq. 8) (Lubner et al., 2017).


(8) 2NADPH+NAD++2Fd=2NADP++NADH+2Fd+-H+

Besides from the Archaeon *Pyrococcus furiosus* (Lubner et al., 2017), Nfn has also been characterized from the bacteria *C. kluyveri* (Wang et al., 2010), *Thermotoga maritima* (Demmer et al., 2015) and *Sporomusa ovata* (Kremp et al., 2020). The enzyme acts as transhydrogenase and in the reverse direction ensures with the “energy rich” Fd^–^ that the NAD^+^/NADPH ratio is kept low at *E*’ = −380 mV and the NAD^+^/NADH ratio high at *E*’ = −280 mV. Hence *in vivo* NADPH is a better reductant and NAD^+^ a better oxidant.

According to Eq. 7, the one-electron reduction potentials of β-FADH^–^ are calculated with an assumed log *K*_*s*_ = −15.2 to ± 0.0295 × (−15.2) + (−0.271) = ± 0.448−0.271 = +0.177 and −0.719 Volt (Figure 3; Buckel and Thauer, 2018a). The electron with the higher potential goes endergonically from +177 mV to α-FAD^–^ (−36 mV) and the lower potential electron goes exergonically from −719 mV to ferredoxin (−390 mV) sitting about 6 Å apart from β-FAD. Thus the two one-electron transfers are coupled; the first electron moves uphill only if the second electron falls downhill to ferredoxin and vice versa (Figure 3). Now the formed α-FADH^–^ rotates by 90° toward Bcd and transfers one electron to δ-FAD of Bcd and the regenerated α-FAD^–^ returns to β-FAD. Surprisingly, in the presence of Bcd, the reduction potential of the formed α-FADH^–^ changes to close to the two-electron reduction potential of α-FAD/α-FADH^–^ = −228 mV, which enables much better electron transfer to the potential of δ-FAD/δ-FAD^–^ = +162 mV. The next electron bifurcation transfers one electron via α-FADH^–^ to δ-FAD^–^ (−63 mV) and the formed δ-FADH^–^ changes to the two-electron reduction potential of −100 mV, which easily reduces crotonyl-CoA to butyryl-CoA (−10 mV; Figure 3).

Probably the reader wonders why during electron bifurcation, α-FAD^•–^ rather than α-FAD acts as high potential acceptor. There are three reasons, firstly the reduction potential of α-FAD with +134 mV is too high, whereas that of α-FAD^•–^ is more in the right range of −36 mV. In electron bifurcation, the potential difference between β-FADH^–^ and α-FAD^•–^ with + 179−(−36) = +215 mV should be similar to the negative difference between the potentials of β-FADH^–^ and Fd with − 719−(−390) = −329 mV, to ensure tight coupling. With α-FAD as acceptor, the difference of + 179−(+134) = +45 mV would be much too low. Secondly, the change of the one-electron reduction potential of α-FADH^–^ to the two-electron reduction potential cannot occur at the state of α-FAD^•–^. Thirdly, as soon as EtfAB encounters NADH, either in vitro or in vivo, α-FAD is reduced to the semiquinone (Sucharitakul et al., 2021a). Finally, stopped flow measurements by Jeerus Sucharitakul directly showed that Etf with α-FADH^–^ indeed transferred one electron to Bcd, whereas with α-FAD^•–^ no electron transfer was observed (Sucharitakul et al., 2021b, unpublished).

## Fermentation of Glutamate: 2-Hydroxyglutarate or 3-Methylaspartate Pathway?

The previous chapters described enzymatic systems which are able to establish electrochemical Na^+^ or H^+^ gradients for energy conservation in anaerobic bacteria. In addition, electron bifurcation as a major source of reduced ferredoxin has been presented. This chapter shows how the two pathways of glutamate fermentation apply these systems to obtain the theoretical yield of ATP/glutamate (eq. 9). −314 kJ/5 glutamate: −66 kJ/ATP = 4.8 ATP/5 glutamate; 0.96 ATP/glutamate.


(9) 5Glutamate+-6HO2+2H→+5NH+4+5CO2+6acetate+-2butyrate+-H2

(Buckel, 2001b).

**FIGURE 4:**
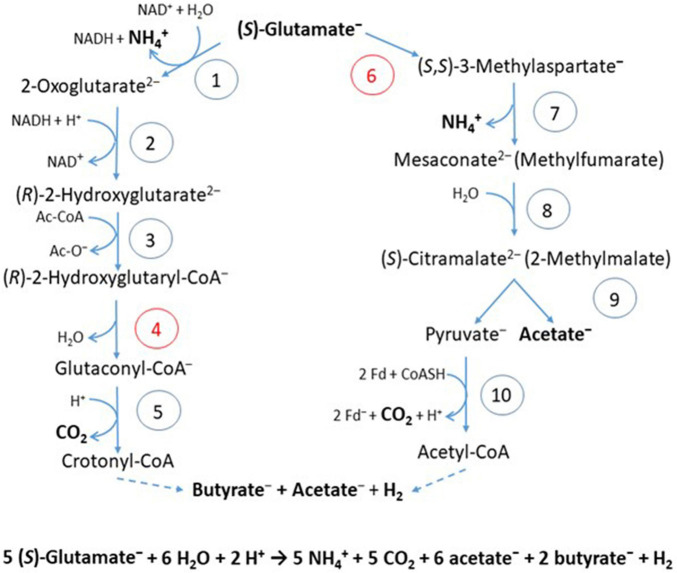
Fermentation of glutamate via 2-hydroxyglutarate or via 3-methylaspartate. The numbers in the circles represent the corresponding enzymes; red numbers denote radical enzymes: 1, (*S*)-glutamate dehydrogenase; 2, (*R*)-2-hydroxyglutarate dehydrogenase; 3, glutaconate CoA-transferase; 4, (*R*)-2-hydroxyglutaryl-CoA dehydratase; 5, glutaconyl-CoA decarboxylase, Na^+^-pumping; 6, glutamate mutase, coenzyme B_12_-dependent; 7, methylaspartase; 8, mesaconase; 9, (*S*)-citramalate lyase; 10, pyruvate:ferredoxin oxidoreductase (PFOR). Ac-CoA, acetyl or glutaconyl-CoA; Fd, ferredoxin; Fd^–^, reduced ferredoxin. The formation of butyrate and acetate are shown in Figure 5. Hydrogen, H_2_ is formed from 2 H^+^ and 2 Fd^–^, catalyzed by a [FeFe]-hydrogenase.

**FIGURE 5:**
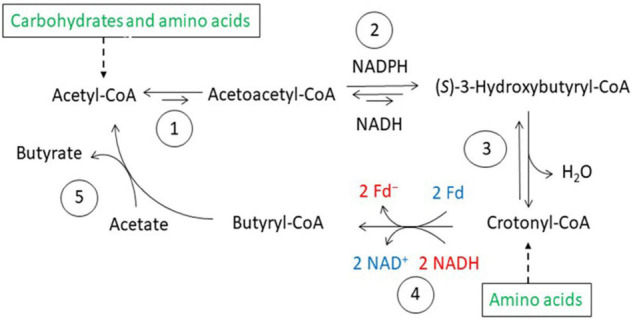
Butyrate synthesis in anaerobic bacteria. 1, Thiolase; 2, (*S*)-3-hydroxybutyryl-CoA dehydrogenases, NADH and NADPH specific; 3, (*S*)-3-hydroxybutyryl-CoA dehydratase; 4, electron bifurcating EtfAB-butyryl-CoA dehydrogease complex; 5, butyrate CoA-transferase.

In the 2-hydroxyglutarate pathway (Figure 4), which has been found in *A. fermentans*, *C. symbiosum*, *P. asaccharolyticus*, and *F. nucleatum* (Buckel and Barker, 1974), NAD^+^ oxidizes glutamate to 2-oxoglutarate and ammonia (Hornby and Engel, 1984). The formed NADH reduces 2-oxoglutarate to (*R*)-2-hydroxyglutarate (Yu et al., 2012), which is converted to the thioester by acetyl-CoA (Buckel et al., 1981) and dehydrated to glutaconyl-CoA; for a review see Buckel (2019). Glutaconyl-CoA is the substrate of the third group of biotin-containing and Na^+^-dependent decarboxylases (Buckel and Semmler, 1983). Glutaconyl-CoA is a vinylogous malonyl-CoA, in which the δ-carboxylate is equally activated as the β-carboxylates in malonyl-CoA and methylmalonyl-CoA. Decarboxylation of 5 glutaconyl-CoA yields 5 × 2 ΔμNa^+^ and 5 crotonyl-CoA (2-butenoyl-CoA), three of which disproportionate oxidatively to 6 acetyl-CoA and 3 NADH (Figure 5). Four NADH reduce 2 crotonyl-CoA to 2 butyryl-CoA and 4 ferredoxin (Fd) to 4 Fd^–^ via electron bifurcation. Two Fd^–^ give rise to H_2_ catalyzed by a [FeFe]hydrogenase and the other two Fd^–^ to 2 ΔμNa^+^ + 1 NADH via Rnf. In summary, the 5 ΔμNa^+^ used for the transport of 5 glutamates into the cell have to be considered (Brüggemann et al., 2003). The net conserved energy amounts to 5 × 2 + 2 – 5 = 7 ΔμNa^+^ and 2 butyryl-CoA + 6 acetyl-CoA, 5 of which are required for the activation of 5 × 2-hydroxyglutarates. The remaining 3 thioesters are equivalent to 3 ATP by SLP (Table 1). If 4 ΔμNa^+^ give 1 ATP, then the yield is 7/4 + 3 = 4.75 (≈ 4.8) ATP/5 glutamate, as expected.

**FIGURE 6:**
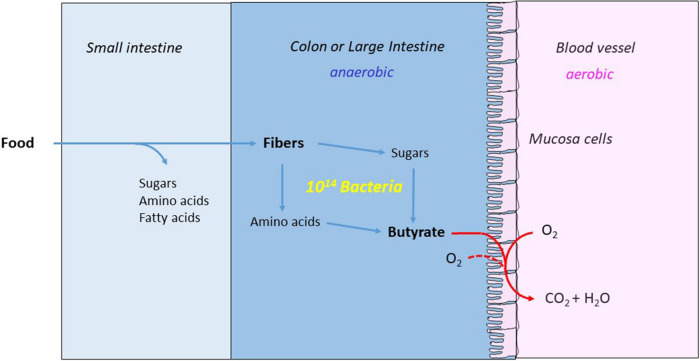
Butyrate nourishes the mucosa cells and makes the colon anaerobic. The major part of the food is digested and absorbed in the small intestine. The remaining fibers are hydrolyzed to monosaccharides and amino acids by the anaerobic bacteria and fermented the large intestine or colon to acetate, propionate and butyrate. The mucosa cells combust the produced butyrate with oxygen from blood and colon, whereby the colon becomes strictly anaerobic.

**TABLE 1:** Energy conservation in the two pathways of glutamate fermentation.

**Path-way**	**ΔμNa^+^ consumed**	**ΔμNa^+^ generated**	**Thioester (TE) consumed**	**TE generated**	**Fd reduced to Fd^–^**	**Fd^–^ oxidized to Fd**	**Sum (generated)**
HG	5 × 1 Transport	5 × 2 by DC 2 by Rnf	5 AcCoA	6 AcCoA 2 BuCoA	4 by EB	2 by Rnf 2 by Hase	7 ΔμNa^+^ 3 TE
MA	5 × 1 Transport	6 × 2 by Rnf	4 AcCoA	5 AcCoA 2 BuCoA	10 by PFOR 4 by EB	12 by Rnf 2 by Hase	7 ΔμNa^+^ 3 TE

*HG, 2-hydroxyglutarate pathway; MA, 3-methylaspartate pathway; DC, Na^+^ pumping decarboxylase; AcCoA, acetyl-CoA; BuCoA, butyryl-CoA; Fd and Fd^–^, oxidized and reduced ferredoxin; EB, electron bifurcation; PFOR, pyruvate ferredoxin oxidoreductase; Hase, [FeFe]hydrogenase.*

Several clostridia, *Clostridium tetanomorphum*, *C. cochlearium*, and *C. tetani*, use a completely different pathway of glutamate fermentation, but with the same stoichiometry as that via 2-hydroxyglutarate (eq. 9) (Buckel and Barker, 1974). This 3-methylaspartate pathway is initiated by the coenzyme B_12_-dependent carbon skeleton rearrangement of (*S*)-glutamate to (2*S*,3*S*)-3-methylaspartate (Barker et al., 1964), from which ammonia is readily eliminated affording mesaconate (methylfumarate) (Barker et al., 1959). Addition of water yields (*S*)-citramalate (2-methylmalate) (Blair and Barker, 1966), which is cleaved to acetate and pyruvate (Buckel and Bobi, 1976). Oxidation of 5 pyruvate to 5 acetyl-CoA, 5 CO_2_ and 10 Fd^–^ is catalyzed by pyruvate:ferredoxin oxidoreductase (PFOR) (Figure 4). 4 Acetyl-CoA condense to 2 acetoacetyl-CoA, which are reduced with 2 NADH to 2 (*S*)-3-hydroxybutyryl-CoA, dehydrated to 2 crotonyl-CoA and further reduced with 4 NADH to 2 butyryl-CoA and 4 reduced ferredoxin via electron bifurcation (Figure 5). For the reduction of 6 NAD^+^ by Rnf, 12 Fd^–^ are consumed and give rise to 12 ΔμNa^+^. The remaining 2 Fd^–^ are used to produce 1 H_2_. The import of 5 glutamate consumes 5 ΔμNa^+^. In summary, 3 ATP are obtained via SLP from 1 acetyl-CoA and 2 butyryl-CoA and 7/4 ATP from the remaining 12–5 = 7 ΔμNa^+^, altogether 4.75 ATP/5 glutamate, the same as in the 2-hydroxyglutarate pathway (Table 1).

The fermentations of glutamate via methylaspartate or 2-hydroxyglutarate lead to identical products and to identical amounts of conserved ATP. Therefore, the question arises why bacteria exclusively use one of these pathways. One would expect that bacteria prefer the 2-hydroxyglutarate pathway because the 3-methylaspartate pathway requires about 30 additional enzymes for the anaerobic biosynthesis of the coenzyme B_12_ (Moore and Warren, 2012), whereas in the 2-hydroxyglutarate pathway only the ubiquitous cofactors NAD, FAD, CoA, pyridoxal-5′-phosphate and biotin are involved. These cofactors are not pathway specific, because they are also necessary for anabolism. In absence of vitamin B_12_, *Fusobacterium varium* uses the 2-hydroxyglutarate pathway, whereas after addition of 1 μM vitamin B_12_ or just 1 μM CoCl_2_ to the medium, the organism ferments glutamate via 3-methylaspartate (Ramezani et al., 2011). Hence, *F. varium* is able to synthesize coenzyme B_12_ when Co^2+^ is present and the argument that the expression of 30 additional genes for B_12_ synthesis favors the 2-hydroxyglutarate pathway does not apply. Therefore, the reason for the existence of this pathway must be found somewhere else.

The organisms using the 2-hydroxyglutarate pathway, *A. fermentans, P. asaccharolyticus, C. symbiosum, F. nucleatum, and F. varium* were isolated from anaerobic niches of the human host, preferentially from the human large intestine. In contrast, the members of the 3-methylaspartate pathway *C. tetanomorphum, C. tetani, C. cochlearium, C. lentoputrescens, C. limosum, C. malenominatum* have been detected outside the gut on places like soil, where organic matter decomposes anaerobically (Buckel, 2001b). The employment of the 2-hydroxyglutarate pathway in the human gut could be due to the much lower oxygen concentration as compared to soil which encounters often exposures to air through plant roots, earthworms, moles, etc. Probably, the oxygen sensitivity of enzymes of an important pathway might be responsible for the ecology of the organism.

Both glutamate fermenting pathways contain oxygen sensitive radical enzymes. Whereas coenzyme B_12_-dependent glutamate mutase in the 3-methylaspartate pathway is only moderately oxygen sensitive, 2-hydroxyglutaryl-CoA dehydratase in the 2-hydroxyglutarate pathway immediately becomes inactive after exposure to air. Coenzyme B_12_-dependent mutases catalyze the reversible radical rearrangement of a methine radical to a methylene radical. The process is initiated by homolysis of the carbon-cobalt bond of coenzyme B_12_. The formed 5′-deoxyadenosyl radical abstracts the *Si*-hydrogen atom from the methylene group at C3 of (*S*)-glutamate and after rearrangement adds it back to the formed methylene radical yielding the methyl group of (2*S*,3*S*)-3-methylaspartate. After each turnover, the radical disappears by reformation of the carbon-cobalt bond of the coenzyme, for a review see Buckel and Golding (1996). Thus, in the presence of air, the radicals are only transiently exposed to oxygen as the coenzyme B_12_-dependent methylmalonyl-CoA mutase in human mitochondria.

In contrast, 2-hydroxyglutaryl-CoA dehydratase acts with a completely different radical mechanism (Buckel and Keese, 1995), for a review see Buckel (2019). The enzyme system is composed of two proteins. The homodimeric activator contains one ADP in each subunit and one [4Fe-4S] cluster coordinated by four cysteines, two from each subunit, similar to the iron protein (NifH) of nitrogenase (Locher et al., 2001). The heterodimeric dehydratase holds one [4Fe-4S] cluster in each subunit. Each cluster is coordinated by three cysteines; the fourth coordination is occupied in subunit A by a sulfur atom and in subunit B by water, which can be replaced by the thioester carbonyl of the substrate. The dehydration is initiated by ATP/ADP exchange of the activator and reduction of its [4Fe-4S]^2+^ cluster by reduced ferredoxin (Fd^–^). Driven by ATP hydrolysis, the extra electron is transferred to or “shot into” the [4Fe-4S] cluster of subunit A and further shifted to cluster B, where 2-hydroxyglutaryl-CoA is bound. The electron in cluster B reduces the thioester carbonyl to a ketyl radical, which due to its lower basicity is replaced from the iron by the hydroxyl group at C-2, a process called ligand swapping. Aided by the iron of cluster B, the nucleophilic ketyl eliminates the hydroxyl group to form an enoxy radical. The hydroxyl group at the iron of the cluster acts as base to remove the now acidic β-proton at C-3. The resulting allylic ketyl replaces the formed water by a second ligand swapping and returns the electron via cluster B to cluster A. The formed product glutaconyl-CoA is released and cluster B is able to accept the next substrate together with the electron form cluster A. The dehydratase is able to catalyze at least 1000 turnovers before another ATP molecule has to be hydrolyzed to continue catalysis (Kim et al., 2005). Proof of this mechanism was obtained by detection of the corresponding allylic ketyl radical of (*R*)-2-hydroxyisocaproyl-CoA dehydratase from *C. difficile* (Kim et al., 2008).

It has been shown experimentally that (*R*)-2-hydroxyacyl-CoA dehydratases are indeed much more oxygen sensitive than coenzyme B_12_-dependent carbon skeleton mutases. Whereas 2-hydroxyglutarate dehydratases had to be purified and assayed under strict exclusion of oxygen (Kim et al., 2005), glutamate mutase could be purified under air. During catalysis, however, after about 1 min a significant effect of air on the rate of 3-methylaspartate formation from glutamate was observed (Leutbecher et al., 1992). In the dehydratases, the electron never disappears during the catalytic cycle and the iron-sulfur cluster of the activator is solvent accessible, which makes it extremely oxygen sensitive. The question arises why the gut, where (*R*)-2-hydroxyacyl-CoA dehydratases are active, contains such low oxygen concentrations despite its ample supply with oxygen-rich blood (see section “Butyrate Provides Anaerobiosis in the Gut”).

## Oxygen-Tolerant and -Intolerant Radical Enzymes

In the previous chapters it has been shown that the 3-methylaspartate pathway tolerates oxygen because after each turnover of glutamate mutase, the cobalt-carbon bond between the 5′-deoxyadenosyl radical and cob(II)alamin is reformed. Thus, only during catalysis the radical is exposed to oxygen. In contrast, in (*R*)-2-hydroxyglutaryl-CoA dehydratase of the alternative glutamate fermenting pathway, the radical is always present and exposed to the medium. In addition, the iron-sulfur clusters of the dehydratase and its activator are very oxygen-sensitive. Most likely, this is the reason, why organisms of the 2-hydroxyglutarate pathway are only found in the gut or in strictly anaerobic marine sediments. Furthermore, B_12_-dependent carbon skeleton mutases and eliminases as well as thiamin diphosphate dependent enzymes are substituted in the gut by glycyl radical enzymes (Table 2). Similar to (*R*)-2-hydroxyacyl-CoA dehydratases, glycyl radical enzymes also require specific activating enzymes. These activases belong to the large family of radical SAM enzymes, which catalyze the one electron-reduction of S-adenosylmethionine (SAM) to methionine and the 5′-deoxyadenosyl radical. This radical irreversibly abstracts one hydrogen from a conserved glycine residue of the enzyme to give a stable protein-bound radical. Upon binding of substrate, the glycyl radical abstracts the sulfhydryl hydrogen form a nearby cysteine residue, which in turn removes the hydrogen atom from the substrate. After the rearrangement, the radical returns to the glycine residue and remains stable until the next turnover, unless it is attacked by oxygen (Knappe and Sawers, 1990; Shisler and Broderick, 2014).

**TABLE 2:** Pairs of oxygen-tolerant and intolerant enzymes which catalyze the same reaction or are key enzymes of alternative pathways leading to the same products.

**Pathway/Enzymes**	**Oxygen-tolerant**	**Oxygen-intolerant**
Glutamate fermentation	Glutamate mutase B_12_	(*R*)-2-Hydroxyglutaryl-CoA dehydratase
Lactate, pyruvate, alanine, cysteine and serine fermentation to propionate	Methylmalonyl-CoA mutase B_12_	(*R*)-Lactyl-CoA dehydratase
Glycerol dehydratase	B_12_-dependent eliminase	Glycyl radical enzyme
Propane-1,2-diol dehydratase	B_12_-dependent eliminase	Glycyl radical enzyme
Ribonucleotide reductase	Class II: B_12_-dependent eliminase and reductase	Class III: Glycyl radical eliminase and reductase
Ethanolamine ammonia lyase	B_12_-dependent eliminase	−
Choline-trimethylamine lyase	−	Glycyl radical enzyme
Threonine and methionine fermentation to propionate and butyrate	TDP-dependent oxidation of 2-oxobutyrate to propionyl-CoA	Dehydration of (*R*)-2-hydroxybutyryl-CoA by (*R*)-lactyl-CoA dehydratase
Oxidation of 2-oxoacids to acyl-CoA	TDP-dependent enzymes: Pyruvate ferredoxin oxidoreductase; pyruvate dehydrogenase (NAD)	Glycyl radical enzyme: pyruvate:formate lyase (PFL)
Taurine fermentation	TDP-dependent enzyme: Sulfoacetaldehyde desulfonase	Glycyl radical enzyme: Isethionate lyase

Many organisms including humans are able to degrade propionate via carboxylation of propionyl-CoA to (*S*)-methylmalonyl-CoA and racemization to (*R*)-methylmalonyl-CoA, which is rearranged to succinyl-CoA mediated by coenzyme B_12_. The Krebs cycle converts succinyl-CoA to oxaloacetate, which enters gluconeogenesis or is degraded via pyruvate to acetyl-CoA. Propionibacteria use the reverse pathway to produce propionate from succinate. *Veillonella alcalescens* and *P. modestum* conserve energy with the biotin-dependent Na^+^-pump (*S*)-methylmalonyl-CoA decarboxylase (Hilpert and Dimroth, 1982; Schink, 1982). In contrast, *Clostridium propionicum*, isolated from marine mud (Cardon and Barker, 1946), converts alanine, cysteine and serine via pyruvate (Hofmeister et al., 1993) to (*R*)-lactate, which is dehydrated via (*R*)-lactyl-CoA to acrylyl-CoA (Schweiger and Buckel, 1984; Hofmeister and Buckel, 1992) and reduced to propionyl-CoA (Hetzel et al., 2003). Like 2-hydroxyglutaryl-CoA dehydratase, lactyl-CoA dehydratase is an extremely oxygen sensitive radical enzyme (Parthasarathy et al., 2010). The rumen micro-organism *Megasphaera elsdenii* reduces about half of the consumed lactate in the same way as *C. propionicum*; the other half is oxidized to acetyl-CoA, from which butyrate (C2 + C2), valerate (C2 + C3) and caproate (3 × C2) are formed (Lewis and Elsden, 1955).

The pyridoxal-5′-phosphate dependent elimination of water from threonine leads to 2-oxobutyrate and ammonia (Hofmeister et al., 1993). Homocysteine, derived from methionine, suffers a similar elimination to 2-oxobutyrate, ammonia and sulfide. Some anaerobic bacteria oxidize 2-oxobutyrate with ferredoxin to propionyl-CoA catalyzed by a thiamin diphosphate (TDP)-dependent enzyme and excrete propionate. *C. propionicum* reduces 2-oxobutyrate to (*R*)-2-hydroxybutyrate and dehydrates (*R*)-2-hydroxybutyryl-CoA to crotonyl-CoA, catalyzed by (*R*)-lactyl-CoA dehydratase (Hofmeister and Buckel, 1992).

Three eliminations of water from 1,2-diols are known, each of which is catalyzed by two different enzymes, either coenzyme B_12_-dependent or by the much more oxygen-sensitive glycyl radical enzymes (Table 2). The coenzyme B_12_-dependent glycerol dehydratase catalyzes the removal of the central hydroxyl group of glycerol yielding 3-hydroxypropanal (Forage and Foster, 1982), which can be reduced to 1,3-propanediol, a building block for polyesters. The discovery of the glycyl radical dehydratase from *Clostridium butyricum* (O’Brien et al., 2004) though extremely oxygen-sensitive, paved the road to a more economic fermentation of 1,3-propanediol from glycerol, because the already established procedure with the coenzyme B_12_-dependent enzyme required feeding with the precious vitamin B_12_. The mechanism of coenzyme B_12_-dependent eliminations was first studied with propane-1,2-diol dehydratase from *Aerobacter aerogenes* by Janós Rétey and Dulio Arigoni in Switzerland (Rétey et al., 1966) as well as Robert H. Abeles (Zagalak et al., 1966) in the United States. In the human gut, degradation of L-fucose (6-deoxygalactose) and L-rhamnose (6-deoxymannose) affords propane-1,2-diol, which is dehydrated to propanal, catalyzed by the glycyl radical enzyme propane-1,2-diol dehydratase (Levin and Balskus, 2018). The third pair of coenzyme B_12_ and glycyl radical enzymes catalyzing the same reaction are classes II and III of ribonucleotide reductase (Greene et al., 2020). The formation of the conserved thiyl radical in this enzyme is performed in class II by the 5′-deoxyadenosyl radical from coenzyme B_12_ and in class III by the glycyl radical. Abstraction of the 3′-hydrogen of the ribonucleotide by the thiyl radical and deprotonation of the 3′-hydroxyl group affords a nucleophilic ketyl (radical anion), which eliminates the adjacent 2′-hydroxyl group (Buckel, 2019). The reduction of the formed enoxy radical differs between the classes. In class II the reductants are two cysteine residues which form a disulfide, whereas class III uses formate for this purpose.

In the eliminations of ammonia from ethanolamine or trimethylamine from choline, carbon nitrogen bonds are broken. Interestingly, for the deamination of ethanolamine only a coenzyme B_12_-dependent enzyme is known (Shibata et al., 2010), whereas the elimination of trimethylamine from choline is exclusively catalyzed by a glycyl radical enzyme (Craciun et al., 2014). Perhaps the different elimination mechanisms, but not the oxygen sensitivity, are responsible for the kind of radical enzyme applied. It has been proposed theoretically (Buckel, 1996; Feliks and Ullmann, 2012; Kovacevic et al., 2018) and demonstrated experimentally (Levin and Balskus, 2018) that in the dehydration of propane-1,2-diol catalyzed by the glycyl radical enzyme, the hydroxyl group at C2 is eliminated. Coenzyme B_12_-dependent propane-1,2-diol dehydratase and glycerol dehydratase, however, shift the hydroxyl group at C2 to C1, forming propane-1,1-diol or 3-hydroxypropane-1,1-diol which dehydrate to the aldehydes. Because trimethylamine is a better leaving group than ammonia, chemistry might compel choline lyase to use the glycyl radical mechanism. Trimethylamine (TMA) is of medical importance, because in the liver it is oxidized to the N-oxide (TMAO), which may cause cancer and other diseases (Gatarek and Kaluzna-Czaplinska, 2021).

The oxidation of pyruvate to acetyl-CoA is a complex reaction for which nature has developed thiamin diphosphate, which causes Umpolung of the carbonyl group of the 2-oxo acid to enable decarboxylation. Most likely a concealed radical is involved in the reaction (Chen et al., 2018). The oxidant is either NAD^+^ or ferredoxin. In aerobes with NAD^+^ the reaction is irreversible, but reversible with ferredoxin in anaerobes (Eqs. 10, 11). Thus this reaction is an example of anaerobes being more efficient energy converters than aerobes.


(10) Pyruvate+-NAD++CoASH→Acetyl-SCoA+NADH+CO;2ΔG°=′-32kJ/mol

(11) Pyruvate+-2Fd+CoASH=Acetyl-SCoA+2Fd+-CO2+H;+ΔG°=′-13kJ/mol

Under strict anaerobic conditions, pyruvate dehydrogenase of *E. coli* (Eq. 10) is replaced by the glycyl radical enzyme, pyruvate formate lyase (PFL), which catalyzes the reversible cleavage of pyruvate to acetyl-CoA and formate (Eq. 12). The enzyme is also present in other enterobacteria and several clostridia.


(12) Pyruvate+-CoASH=Acetyl-SCoA+formate;-ΔG°=′-19kJ/mol

The degradation of taurine (2-aminoethylsulfonate) in aerobes and facultative anaerobes affords sulfoacetaldehyde by amino transfer to pyruvate. The aldehyde is converted by the TDP-dependent sulfoacetaldehyde acetyltransferase Xsc to acetyl phosphate and sulfite (Ruff et al., 2003). Under the strict anaerobic conditions in the gut, the Bacillus *Bilophila wadsworthia* bypasses Xcs with an aldehyde reductase and the glycyl radical enzyme isethionate lyase. This enzyme catalyzes the radical diol dehydratase-like elimination of isethionate (2-sulfoethanol) yielding acetaldehyde and sulfite which is reduced to H_2_S (Peck et al., 2019). AdhE catalyzes the oxidation of acetaldehyde to acetyl-CoA, which as well as acetyl phosphate yields ATP by SLP. The genes *iseG* and *iseH* coding for isethionate lyase and its activating enzyme were also detected in several well-known sulfate reducing organisms (Dawson et al., 2021). The genes from *Desulfovibrio vulgaris* str. Hildenborough were expressed in *E. coli* and the produced proteins characterized as active isethionate lyase (Xing et al., 2019; Wei and Zhang, 2021).

In this review it has become apparent that radical reactions play an important part in the metabolism of anaerobes and most likely already in prebiotic chemistry (Dragicevic et al., 2015). Table 2 summarizes the pairs of oxygen-tolerant and intolerant enzymes which catalyze radical reactions leading to the same product or are key enzymes of alternative pathways. Whereas the oxygen-intolerant 2-hydroxyacyl-CoA dehydratases and glycyl radical enzymes have simple cofactors, the oxygen-tolerant coenzyme B_12_ and TDP-dependent enzymes use the most complex cofactors known in biochemistry (Jurgenson et al., 2009; Moore and Warren, 2012). Apparently, the oxygen-intolerant radical enzymes evolved first and prevailed under the primeval atmosphere until the oxygen concentration raised, which forced nature to develop the oxygen tolerant and much more complex cofactors coenzyme B_12_ and TDP. The oxygen-intolerant radical enzymes survived only in strictly anaerobic places such as the human gut and marine sediments. Probably, these coenzyme B_12_ and TDP dependent fermentations were not present at the origin of life, but emerged just before or during the Great Oxidation event at 2.1 billion years ago (Anbar et al., 2007).

## Butyrate Provides Anaerobiosis in the Gut

Many obligate anaerobic bacteria in the human gut produce butyrate. The most common pathway is the condensation of two acetyl-CoA to acetoacetyl-CoA, followed by the first reduction to (*S*)-3-hydroxybutyryl-CoA, dehydration to crotonyl-CoA and the second reduction to butyryl-CoA as shown in the fermentation of glutamate via 3-methylaspartate. The first reductants are NADH or at low acetyl-CoA concentrations NADPH as found in *Clostridium kluyveri*. The stronger reductant NADPH helps to shift the unfavorable equilibrium of acetyl-CoA condensation to acetoacetyl-CoA toward (*S*)-3-hydroxybutyryl-CoA (Figure 5). The second reduction by NADH is coupled to the reduction of ferredoxin via electron bifurcation (Buckel and Thauer, 2013). Butyrate is released either by a CoA-transferase with acetate or by SLP via butyryl-phosphate (Louis and Flint, 2017). This pathway is used by most organisms fermenting carbohydrates, as are bacteria related to *Roseburia intestinalis*, *Faecalibacterium prausnitzii*, and *Eubacterium hallii* (all belong to Firmicutes). They are among the most abundant known butyrate-producing bacteria in human feces, comprising about 8% of the total flora (Hold et al., 2003). The majority of the bacteria in the human gut belong to Bacteroidetes (Gevers et al., 2012; Huttenhower and Gevers, 2012), which produce acetate and succinate rather than butyrate. As mentioned above *A. fermentans*, *M. elsdenii*, and *C. difficile* also use electron bifurcation for butyrate synthesis.

To the author’s knowledge, electron bifurcation with crotonyl-CoA appears to be the only pathway to butyrate in anaerobes. Probably no anaerobe wants to abandon the large energy released during butyrate synthesis by taking another route. Some acetate assimilating aerobes, however, reduce crotonyl-CoA with NADH directly to butyryl-CoA without proceeding via Etf. Actually the enzyme acts as crotonyl-CoA carboxylase yielding ethylmalonyl-CoA. Only at low CO_2_ concentrations the product is butyryl-CoA (Erb et al., 2007).

Fermentations of amino acids, which give rise to butyrate, are degraded to acetyl-CoA or directly to crotonyl-CoA as shown by the fermentation of glutamate via 2-hydroxyglutarate. *Clostridium propionicum* oxidizes threonine and methionine to (*R*)-2-hydroxybutyrate which is converted to crotonyl-CoA by a mechanism similar to that shown for (*R*)-2-hydroxyglutarate (Barker and Wiken, 1948). The complex anaerobic degradation of lysine in *Clostridium subterminale* SB4 (Barker, 1981) and *Fusobacterium nucleatum* (Barker et al., 1982) involves the radical SAM-dependent 2,3-shift and the coenzyme B_12_-dependent 6,5-shift of the amino groups to 3,5-diaminohexanoate (Ruzicka et al., 2000). The latter is deaminated to 3-oxo-5-aminohexanoate and cleaved with acetyl-CoA to acetoacetate and 3-aminobutyryl-CoA followed by deamination to crotonyl-CoA (Jeng and Barker, 1974) and reduction to butyryl-CoA, which forms butyrate and acetoacetyl-CoA with acetoacetate. Histidine is degraded to butyrate via glutamate either by *C. tetanomorphum* via the 3-methylaspartate pathway or by *C. symbiosum* via the 2-hydroxyglutarate pathway (Barker, 1981). In a Stickland type fermentation, proline is reduced to 5-aminovalerate by *Clostridium sporogenes* (Stickland, 1935), which is deaminated to 5-hydroxyvalerate and further converted via homocrotonyl-CoA to valerate (homobutyrate) by *Clostridium viride* (Eikmanns and Buckel, 1991; Buckel et al., 1994).

In the human gut, butyrate is the preferred nutrient of the mucosa cells, which form the inner wall of the large intestine, also called colonic epithelia cells. Butyrate activates the nuclear peroxisome proliferator-activated receptor-γ (PPAR-γ), a transcription factor, which in turn stimulates mitochondrial β-oxidation. The very active β-oxidation of butyrate is fueled by oxygen from the blood and consumes any remaining oxygen inside of the intestine resulting in an oxygen-free medium, where anaerobes optimally thrive. PPAR-γ also inhibits expression of the *NO2* gene, which codes for an enzyme that catalyzes the oxidation of arginine with oxygen to nitric oxide (NO). NO is further oxidized to nitrate, an electron acceptor for potentially pathogenic enterobacteria. For example, *E. coli*, the best known bacterium of the human gut, is well adapted to the strict anaerobic environment as demonstrated by its glycyl radical enzymes class III ribonucleotide reductase and pyruvate:formate lyase (PFL, eq. 11). However, in the presence of oxygen or nitrate the facultative anaerobe forms pyruvate dehydrogenase (eq. 10) and multiplies very fast, from about 1% of the anaerobic microbiome to a major inhabitant of the gut. In case, the quickly spreading *E. coli* strain is pathogenic, severe diseases can occur. To prevent such events, butyrate nourishes colonic epithelia cells, which provides an oxygen-free medium for the microbiome in the large intestine and protects the host from harmful bacteria (Cani, 2017). For a constant butyrate production, the microbiome must be well fed with carbohydrates and amino acids. However, glucose, lactose, starch, glycogen and most proteins are digested and resorbed already in the small intestine, and nothing is left for the microbiome in the gut. Therefore, the human diet must contain indigestible carbohydrate and protein fibers for the mircrobiome, which is able to hydrolyze almost every glycosidic and peptide bond. Hence, fibers for the human diet are necessary for a healthy gut. The microbiologists Erica and Justin Sonnenburg remind their children to eat enough fibers by saying “Feed your bugs!” (Sonnenburg and Sonnenburg, 2015).

## Author Contributions

The author confirms being the sole contributor of this work and has approved it for publication.

## Conflict of Interest

The author declares that the research was conducted in the absence of any commercial or financial relationships that could be construed as a potential conflict of interest.

## Publisher’s Note

All claims expressed in this article are solely those of the authors and do not necessarily represent those of their affiliated organizations, or those of the publisher, the editors and the reviewers. Any product that may be evaluated in this article, or claim that may be made by its manufacturer, is not guaranteed or endorsed by the publisher.

## Abbreviations

FAD^•–^: FAD anionic semiquinone
FADH^–^: FAD hydroquinone
Fd: ferredoxin
Fd^–^: reduced ferredoxin.

## References

[B1] Anbar A. D. Duan Y. Lyons T. W. Arnold G. L. Kendall B. Creaser R. A. (2007). A whiff of oxygen before the great oxidation event? *Science* 317 1903–1906. 10.1126/science.1140325 17901330

[B2] Barker H. A. (1981). Amino acid degradation by anaerobic bacteria. *Annu. Rev. Biochem.* 50 23–40. 10.1146/annurev.bi.50.070181.000323 6791576

[B3] Barker H. A. Kahn J. M. Hedrick L. (1982). Pathway of lysine degradation in *Fusobacterium nucleatum*. *J. Bacteriol.* 152 201–207. 10.1128/jb.152.1.201-207.1982 6811551 PMC221392

[B4] Barker H. A. Smyth R. D. Wilson R. M. Weissbach H. (1959). The purification and properties of beta-methylaspartase. *J. Biol. Chem.* 234 320–328. 10.1016/s0021-9258(18)70297-4 13630903

[B5] Barker H. A. Suzuki F. Iodice A. Rooze V. (1964). Glutamate mutase reaction. *Ann. N. Y. Acad. Sci.* 112 644–654. 10.1111/j.1749-6632.1964.tb45041.x 14167298

[B6] Barker H. A. Wiken T. (1948). The origin of butyric acid in the fermentation of threonine by *Clostridium propionicum*. *Arch. Biochem.* 17 149–151. 18910688

[B7] Bergdoll L. Ten Brink F. Nitschke W. Picot D. Baymann F. (2016). From low- to high-potential bioenergetic chains: thermodynamic constraints of Q-cycle function. *Biochim. Biophys. Acta* 1857 1569–1579. 10.1016/j.bbabio.2016.06.006 27328272

[B8] Biegel E. Müller V. (2010). Bacterial Na^+^-translocating ferredoxin:NAD^+^ oxidoreductase. *Proc. Natl. Acad. Sci. U.S.A.* 107 18138–18142. 10.1073/pnas.1010318107 20921383 PMC2964206

[B9] Blair A. H. Barker H. A. (1966). Assay and purification of (+)-citramalate hydro-lyase components from *Clostridium tetanomorphum*. *J. Biol. Chem.* 241 400–408. 10.1016/s0021-9258(18)96931-0 5903732

[B10] Boiangiu C. D. Jayamani E. Brügel D. Herrmann G. Kim J. Forzi L. (2005). Sodium ion pumps and hydrogen production in glutamate fermenting anaerobic bacteria. *J. Mol. Microbiol. Biotechnol.* 10 105–119. 10.1159/000091558 16645308

[B11] Brüggemann H. Baumer S. Fricke W. F. Wiezer A. Liesegang H. Decker I. (2003). The genome sequence of *Clostridium tetani*, the causative agent of tetanus disease. *Proc. Natl. Acad. Sci. U.S.A.* 100 1316–1321. 10.1073/pnas.0335853100 12552129 PMC298770

[B12] Buckel W. (1990). “Amino acid fermentation: coenzyme B_12_-dependent and -independent pathways,” in *The Molecular Basis of Bacterial Metabolism 41. Colloquium - Mosbach 1990*, eds Hauska G. Thauer R. (Heidelberg: Springer Verlag), 21–30. 10.1007/978-3-642-75969-7_3

[B13] Buckel W. (1996). Unusual dehydrations in anaerobic bacteria: considering ketyls (radical anions) as reactive intermediates in enzymatic reactions. *FEBS Lett.* 389 20–24. 10.1016/0014-5793(96)00530-3 8682197

[B14] Buckel W. (2001a). Sodium ion-translocating decarboxylases. *Biochim. Biophys. Acta* 1505 15–27. 10.1016/s0005-2728(00)00273-5 11248185

[B15] Buckel W. (2001b). Unusual enzymes involved in five pathways of glutamate fermentation. *Appl. Microbiol. Biotechnol.* 57 263–273. 10.1007/s002530100773 11759672

[B16] Buckel W. (2019). Enzymatic reactions involving ketyls: from a chemical curiosity to a general biochemical mechanism. *Biochemistry* 58 5221–5233. 10.1021/acs.biochem.9b00171 30995029

[B17] Buckel W. Barker H. A. (1974). Two pathways of glutamate fermentation by anaerobic bacteria. *J. Bacteriol.* 117 1248–1260. 10.1128/jb.117.3.1248-1260.1974 4813895 PMC246608

[B18] Buckel W. Bobi A. (1976). The enzyme complex citramalate lyase from *Clostridium tetanomorphum*. *Eur. J. Biochem*. 64 255–262. 10.1111/j.1432-1033.1976.tb10295.x 1278156

[B19] Buckel W. Dorn U. Semmler R. (1981). Glutaconate CoA-transferase from *Acidaminococcus fermentans*. *Eur. J. Biochem.* 118 315–321. 10.1111/j.1432-1033.1981.tb06404.x 6945182

[B20] Buckel W. Golding B. (1996). Glutamate and 2-methyleneglutarate mutase: from microbial curiosities to paradigms for coenzyme B_12_-dependent enzymes. *Chem. Soc. Rev.* 25 329–337. 10.1039/cs9962500329

[B21] Buckel W. Janssen P. H. Schuhmann A. Eikmanns U. Messner P. Sleytr U. (1994). *Clostridium viride* sp. nov., a strictly anaerobic bacterium using 5-aminovalerate as growth substrate, previously assigned to *Clostridium aminovalericum*. *Arch. Microbiol.* 162 387–394.

[B22] Buckel W. Keese R. (1995). One-electron redox reactions of CoASH esters in anaerobic bacteria – a mechanistic proposal. *Angew. Chem.* 34 1502–1506. 10.1002/anie.199515021

[B23] Buckel W. Semmler R. (1982). A biotin-dependent sodium pump: glutaconyl-CoA decarboxylase from *Acidaminococcus fermentans*. *FEBS Lett.* 148 35–38. 10.1016/0014-5793(82)81237-4 6293874

[B24] Buckel W. Semmler R. (1983). Purification, characterisation and reconstitution of glutaconyl-CoA decarboxylase, a biotin-dependent sodium pump from anaerobic bacteria. *Eur. J. Biochem.* 136 427–434. 10.1111/j.1432-1033.1983.tb07760.x 6628393

[B25] Buckel W. Thauer R. K. (2013). Energy conservation via electron bifurcating ferredoxin reduction and proton/Na^+^ translocating ferredoxin oxidation. *Biochim. Biophys. Acta* 1827 94–113. 10.1016/j.bbabio.2012.07.002 22800682

[B26] Buckel W. Thauer R. K. (2018a). Flavin-based electron bifurcation, a new mechanism of biological energy coupling. *Chem. Rev.* 118 3862–3886. 10.1021/acs.chemrev.7b00707 29561602

[B27] Buckel W. Thauer R. K. (2018b). Flavin-based electron bifurcation, ferredoxin, flavodoxin, and anaerobic respiration with protons (Ech) or NAD^+^ (Rnf) as electron acceptors: a historical review. *Front. Microbiol.* 9:401. 10.3389/fmicb.2018.00401 29593673 PMC5861303

[B28] Cani P. D. (2017). Gut cell metabolism shapes the microbiome. *Science* 357 548–549. 10.1126/science.aao2202 28798116

[B29] Cardon B. P. Barker H. A. (1946). Two new amino-acid-fermenting bacteria, *Clostridium propionicum* and *Diplococcus glycinophilus*. *J. Bacteriol.* 52 629–634. 10.1128/jb.52.6.629-634.1946 16561227 PMC518248

[B30] Chen P. Y. Aman H. Can M. Ragsdale S. W. Drennan C. L. (2018). Binding site for coenzyme a revealed in the structure of pyruvate:ferredoxin oxidoreductase from *Moorella thermoacetica*. *Proc. Natl. Acad. Sci. U.S.A.* 115 3846–3851. 10.1073/pnas.1722329115 29581263 PMC5899475

[B31] Chowdhury N. P. Kahnt J. Buckel W. (2015). Reduction of ferredoxin or oxygen by flavin-based electron bifurcation in *Megasphaera elsdenii*. *FEBS J.* 282 3149–3160. 10.1111/febs.13308 25903584

[B32] Chowdhury N. P. Klomann K. Seubert A. Buckel W. (2016). Reduction of flavodoxin by electron bifurcation and sodium ion-dependent reoxidation by NAD^+^ catalyzed by ferredoxin-NAD^+^ reductase (Rnf). *J. Biol. Chem.* 291 11993–12002. 10.1074/jbc.m116.726299 27048649 PMC4933252

[B33] Chowdhury N. P. Mowafy A. M. Demmer J. K. Upadhyay V. Koelzer S. Jayamani E. (2014). Studies on the mechanism of electron bifurcation catalyzed by electron transferring flavoprotein (Etf) and butyryl-CoA dehydrogenase (Bcd) of *Acidaminococcus fermentans*. *J. Biol. Chem.* 289 5145–5157. 10.1074/jbc.m113.521013 24379410 PMC3931072

[B34] Craciun S. Marks J. A. Balskus E. P. (2014). Characterization of choline trimethylamine-lyase expands the chemistry of glycyl radical enzymes. *ACS Chem. Biol.* 9 1408–1413. 10.1021/cb500113p 24854437

[B35] Dawson C. D. Irwin S. M. Backman L. R. F. Le C. Wang J. X. Vennelakanti V. (2021). Molecular basis of C-S bond cleavage in the glycyl radical enzyme isethionate sulfite-lyase. *Cell Chem. Biol.* 28 1–14. 10.1016/j.chembiol.2021.03.001 33773110 PMC8473560

[B36] Demmer J. K. Huang H. Wang S. Demmer U. Thauer R. K. Ermler U. (2015). Insights into flavin-based electron bifurcation via the NADH-dependent reduced ferredoxin:NADP oxidoreductase structure. *J. Biol. Chem.* 290 21985–21995. 10.1074/jbc.m115.656520 26139605 PMC4571952

[B37] Demmer J. K. Pal Chowdhury N. Selmer T. Ermler U. Buckel W. (2017). The semiquinone swing in the bifurcating electron transferring flavoprotein/butyryl-CoA dehydrogenase complex from *Clostridium difficile*. *Nat. Commun.* 8:1577. 29146947 10.1038/s41467-017-01746-3 PMC5691135

[B38] Dimroth P. (1980). A new sodium-transport system energized by the decarboxylation of oxaloacetate. *FEBS Lett.* 122 234–236. 10.1016/0014-5793(80)80446-7 7009209

[B39] Dimroth P. Thomer A. (1986). Kinetic analysis of the reaction mechanism of oxaloacetate decarboxylase from *Klebsiella aerogenes*. *Eur. J. Biochem.* 156 157–162. 10.1111/j.1432-1033.1986.tb09561.x 3082631

[B40] Dimroth P. Thomer A. (1993). On the mechanism of sodium ion translocation by oxaloacetate decarboxylase of *Klebsiella pneumoniae*. *Biochemistry* 32 1734–1739. 10.1021/bi00058a006 8382519

[B41] Djordjevic S. Pace C. P. Stankovich M. T. Kim J. J. (1995). Three-dimensional structure of butyryl-CoA dehydrogenase from *Megasphaera elsdenii*. *Biochemistry* 34 2163–2171. 10.1021/bi00007a009 7857927

[B42] Dragicevic I. Baric D. Kovacevic B. Golding B. T. Smith D. M. (2015). Non-enzymatic ribonucleotide reduction in the prebiotic context. *Chemistry* 21 6132–6143. 10.1002/chem.201405741 25754795

[B43] Eikmanns U. Buckel W. (1991). Crystalline green 5-hydroxyvaleryl-CoA dehydratase from *Clostridium aminovalericum*. *Eur. J. Biochem.* 197 661–668. 10.1111/j.1432-1033.1991.tb15956.x 2029896

[B44] Engel P. C. Massey V. (1971). Green butyryl-coenzyme a dehydrogenase. an enzyme-acyl-coenzyme a complex. *Biochem. J.* 125 889–902. 10.1042/bj1250889 5145911 PMC1178195

[B45] Erb T. J. Berg I. A. Brecht V. Müller M. Fuchs G. Alber B. E. (2007). Synthesis of C5-dicarboxylic acids from C2-units involving crotonyl-CoA carboxylase/reductase: the ethylmalonyl-CoA pathway. *Proc. Natl. Acad. Sci. U.S.A.* 44 31496–31502. 10.1073/pnas.0702791104 PMC1965564 17548827

[B46] Ezaki T. Yamamoto N. Ninomiya K. Suzuki S. Yabuuchi E. (1983). Transfer of *Peptococcus indolicus, Peptococcus asaccharolyticus, Peptococeus prevotii and Peptococcus magnus* to the genus Peptostreptococcus and proposal of *Peptostreptococcus tetardius* sp. nov. *Int. J. Syst. Bacteriol.* 33 683–689. 10.1099/00207713-33-4-683

[B47] Feliks M. Ullmann G. M. (2012). Glycerol dehydratation by the B_12_-independent enzyme may not involve the migration of a hydroxyl group: a computational study. *J. Phys. Chem. B* 116 7076–7087. 10.1021/jp301165b 22626266

[B48] Forage R. G. Foster M. A. (1982). Glycerol fermentation in *Klebsiella pneumoniae*: functions of the coenzyme B_12_-dependent glycerol and diol dehydratases. *J. Bacteriol.* 149 413–419. 10.1128/jb.149.2.413-419.1982 7035429 PMC216523

[B49] Gatarek P. Kaluzna-Czaplinska J. (2021). Trimethylamine N-oxide (TMAO) in human health. *EXCLI J.* 20 301–319. 33746664 10.17179/excli2020-3239 PMC7975634

[B50] Gevers D. Pop M. Schloss P. D. Huttenhower C. (2012). Bioinformatics for the human microbiome project. *PLoS Comput. Biol.* 8:e1002779. 10.1371/journal.pcbi.1002779 23209389 PMC3510052

[B51] Greene B. L. Kang G. Cui C. Bennati M. Nocera D. G. Drennan C. L. (2020). Ribonucleotide reductases: structure, chemistry, and metabolism suggest new therapeutic targets. *Annu. Rev. Biochem.* 98 45–75. 10.1146/annurev-biochem-013118-111843 32569524 PMC7316142

[B52] Hans M. Bill E. Cirpus I. Pierik A. J. Hetzel M. Alber D. (2002). Adenosine triphosphate-induced electron transfer in 2-hydroxyglutaryl-CoA dehydratase from *Acidaminococcus fermentans*. *Biochemistry* 41 5873–5882. 10.1021/bi020033m 11980491

[B53] Herrmann G. (2008). *Enzymes of Two Clostridial Amino-Acid Fermentation Pathways.* Ph. D. thesis. Germany: Philipps Universität Marburg.

[B54] Herrmann G. Jayamani E. Mai G. Buckel W. (2008). Energy conservation via electron-transferring flavoprotein in anaerobic bacteria. *J. Bacteriol.* 190 784–791. 10.1128/jb.01422-07 18039764 PMC2223574

[B55] Hess V. Gallegos R. Jones J. A. Barquera B. Malamy M. H. Müller V. (2016). Occurrence of ferredoxin:NAD(+) oxidoreductase activity and its ion specificity in several Gram-positive and Gram-negative bacteria. *PeerJ* 4:e1515. 10.7717/peerj.1515 26793417 PMC4715464

[B56] Hess V. Schuchmann K. Müller V. (2013). The ferredoxin:NAD^+^ oxidoreductase (Rnf) from the acetogen *Acetobacterium woodii* requires Na^+^ and is reversibly coupled to the membrane potential. *J. Biol. Chem.* 288 31496–31502. 10.1074/jbc.m113.510255 24045950 PMC3814746

[B57] Hetzel M. Brock M. Selmer T. Pierik A. J. Golding B. T. Buckel W. (2003). Acryloyl-CoA reductase from *Clostridium propionicum*. an enzyme complex of propionyl-CoA dehydrogenase and electron-transferring flavoprotein. *Eur. J. Biochem.* 270 902–910. 10.1046/j.1432-1033.2003.03450.x 12603323

[B58] Hilpert W. Dimroth P. (1982). Conversion of the chemical energy of methylmalonyl-CoA decarboxylation into a Na^+^ gradient. *Nature* 296 584–585. 10.1038/296584a0 7070502

[B59] Hilpert W. Schink B. Dimroth P. (1984). Life by a new decarboxylation-dependent energy conservation mechanism with Na^+^ as coupling ion. *EMBO J.* 3 1665–1670. 10.1002/j.1460-2075.1984.tb02030.x 16453537 PMC557580

[B60] Hofmeister A. E. Buckel W. (1992). (*R*)-lactyl-CoA dehydratase from *Clostridium propionicum*. Stereochemistry of the dehydration of (*R*)-2-hydroxybutyryl-CoA to crotonyl-CoA. *Eur. J. Biochem.* 206 547–552. 10.1111/j.1432-1033.1992.tb16958.x 1597194

[B61] Hofmeister A. E. Grabowski R. Linder D. Buckel W. (1993). L-serine and L-threonine dehydratase from *Clostridium propionicum*. two enzymes with different prosthetic groups. *Eur. J. Biochem.* 215 341–349. 10.1111/j.1432-1033.1993.tb18040.x 8344301

[B62] Hold G. L. Schwiertz A. Aminov R. I. Blaut M. Flint H. J. (2003). Oligonucleotide probes that detect quantitatively significant groups of butyrate-producing bacteria in human feces. *Appl. Environ. Microbiol.* 69 4320–4324. 10.1128/aem.69.7.4320-4324.2003 12839823 PMC165216

[B63] Hornby D. P. Engel P. C. (1984). Characterization of *Peptostreptococcus asaccharolyticus* glutamate dehydrogenase purified by dye-ligand chromatography. *J. Gen. Microbiol.* 130 2385–2394. 10.1099/00221287-130-9-2385 6502134

[B64] Huttenhower C. Gevers D. (2012). Structure, function and diversity of the healthy human microbiome. *Nature* 486 207–214. 10.1038/nature11234 22699609 PMC3564958

[B65] Imkamp F. Biegel E. Jayamani E. Buckel W. Müller V. (2007). Dissection of the caffeate respiratory chain in the acetogen *Acetobacterium woodii:* Identification of an Rnf-type NADH dehydrogenase as a potential coupling site. *J. Bacteriol.* 189 8145–8153. 10.1128/jb.01017-07 17873051 PMC2168664

[B66] Jayamani E. (2008). *A Unique Way of Energy Conservation in Glutamate Fermenting Clostridia* Ph D dissertation. Germany: Philipps-University Marburg.

[B67] Jeng I. Barker H. A. (1974). Purification and properties of l-3-aminobutyryl coenzyme a deaminase from a lysine-fermenting *Clostridium*. *J. Biol. Chem.* 249 6578–6584. 10.1016/s0021-9258(19)42195-9 4420467

[B68] Jurgenson C. T. Begley T. P. Ealick S. E. (2009). The structural and biochemical foundations of thiamin biosynthesis. *Annu. Rev. Biochem.* 78 569–603. 10.1146/annurev.biochem.78.072407.102340 19348578 PMC6078420

[B69] Kayastha K. Vitt S. Buckel W. Ermler U. (2021). Flavins in the electron bifurcation process. *Arch. Biochem. Biophys.* 701:108796. 10.1016/j.abb.2021.108796 33609536

[B70] Kim J. Darley D. Buckel W. (2005). 2-Hydroxyisocaproyl-CoA dehydratase and its activator from *Clostridium difficile*. *FEBS J.* 272 550–561. 10.1111/j.1742-4658.2004.04498.x 15654892

[B71] Kim J. Darley D. J. Buckel W. Pierik A. J. (2008). An allylic ketyl radical intermediate in clostridial amino-acid fermentation. *Nature* 452 239–242. 10.1038/nature06637 18337824

[B72] Knappe J. Sawers G. (1990). A radical-chemical route to acetyl-CoA: the anaerobically induced pyruvate formate-lyase system of *Escherichia coli*. *FEMS Microbiol. Rev.* 6 383–398. 10.1111/j.1574-6968.1990.tb04108.x 2248795

[B73] Kovacevic B. Baric D. Babic D. Bilic L. Hanzevacki M. Sandala G. M. (2018). Computational tale of two enzymes: glycerol dehydration with or without B_12_. *J. Am. Chem. Soc.* 140 8487–8496. 10.1021/jacs.8b03109 29894625

[B74] Kremp F. Roth J. Müller V. (2020). The *Sporomusa* type Nfn is a novel type of electron-bifurcating transhydrogenase that links the redox pools in acetogenic bacteria. *Sci. Rep.* 10:14872. 32913242 10.1038/s41598-020-71038-2 PMC7483475

[B75] Kress D. Brügel D. Schall I. Linder D. Buckel W. Essen L. O. (2009). An asymmetric model for Na^+^-translocating glutaconyl-CoA decarboxylases. *J. Biol. Chem.* 284 28401–28409. 10.1074/jbc.m109.037762 19654317 PMC2788889

[B76] Laubinger W. Dimroth P. (1988). Characterization of the ATP synthase of *Propionigenium modestum* as a primary sodium pump. *Biochemistry* 27 7531–7537. 10.1021/bi00419a053 2905167

[B77] Leutbecher U. Böcher R. Linder D. Buckel W. (1992). Glutamate mutase from *Clostridium cochlearium*. purification, cobamide content and stereospecific inhibitors. *Eur. J. Biochem.* 205 759–765. 10.1111/j.1432-1033.1992.tb16840.x 1315276

[B78] Levin B. J. Balskus E. P. (2018). Characterization of 1,2-propanediol dehydratases reveals distinct mechanisms for B_12_-dependent and glycyl radical enzymes. *Biochemistry* 57 3222–3226. 10.1021/acs.biochem.8b00164 29526088

[B79] Lewis D. Elsden S. R. (1955). The fermentation of L-threonine, L-serine, L-cysteine and acrylic acid by a gram-negative coccus. *Biochem. J.* 60 683–692. 10.1042/bj0600683 13249967 PMC1216171

[B80] Li F. Hinderberger J. Seedorf H. Zhang J. Buckel W. Thauer R. K. (2008). Coupled ferredoxin and crotonyl coenzyme A (CoA) reduction with NADH catalyzed by the butyryl-CoA dehydrogenase/Etf complex from *Clostridium kluyveri*. *J. Bacteriol.* 190 843–850. 10.1128/jb.01417-07 17993531 PMC2223550

[B81] Locher K. P. Hans M. Yeh A. P. Schmid B. Buckel W. Rees D. C. (2001). Crystal structure of the *Acidaminococcus fermentans* 2-hydroxyglutaryl-CoA dehydratase component A. *J. Mol. Biol.* 307 297–308. 10.1006/jmbi.2000.4496 11243821

[B82] Louis P. Flint H. J. (2017). Formation of propionate and butyrate by the human colonic microbiota. *Environ. Microbiol.* 19 29–41. 10.1111/1462-2920.13589 27928878

[B83] Lubner C. E. Jennings D. P. Mulder D. W. Schut G. J. Zadvornyy O. A. Hoben J. P. (2017). Mechanistic insights into energy conservation by flavin-based electron bifurcation. *Nat. Chem. Biol.* 13 655–659. 10.1038/nchembio.2348 28394885 PMC7646311

[B84] Marchandin H. Teyssier C. Campos J. Jean-Pierre H. Roger F. Gay B. (2010). *Negativicoccus succinicivorans* gen. nov., sp. nov., isolated from human clinical samples, emended description of the family *Veillonellaceae* and description of *Negativicutes* classis nov., *Selenomonadales* ord. nov. and fam. nov. in the bacterial phylum Firmicutes. *Int. J. Syst. Evol. Microbiol.* 60 1271–1279. 10.1099/ijs.0.013102-0 19667386

[B85] Moore S. J. Warren M. J. (2012). The anaerobic biosynthesis of vitamin B_12_. *Biochem. Soc. Trans.* 40 581–586. 10.1042/bst20120066 22616870

[B86] Moser C. C. Keske J. M. Warncke K. Farid R. S. Dutton P. L. (1992). Nature of biological electron transfer. *Nature* 355 796–802. 10.1038/355796a0 1311417

[B87] Nitschke W. Russell M. J. (2012). Redox bifurcations: mechanisms and importance to life now, and at its origin: a widespread means of energy conversion in biology unfolds. *Bioessays* 34 106–109. 10.1002/bies.201100134 22045626

[B88] O’Brien J. R. Raynaud C. Croux C. Girbal L. Soucaille P. Lanzilotta W. N. (2004). Insight into the mechanism of the B_12_-independent glycerol dehydratase from *Clostridium butyricum*: preliminary biochemical and structural characterization. *Biochemisty* 43 4635–4645. 10.1021/bi035930k 15096031

[B89] Otto R. Sonnenberg A. S. M. Veldkamp H. Konings W. N. (1980). Generation of an electrochemical proton gradient in *Streptococcus cremoris* by lactate efflux. *Proc. Natl. Acad. Sci. U.S.A.* 77 5502–5506. 10.1073/pnas.77.9.5502 6254084 PMC350089

[B90] Parthasarathy A. Buckel W. Smith D. M. (2010). On the thermodynamic equilibrium between (*R*)-2-hydroxyacyl-CoA and 2-enoyl-CoA. *FEBS J.* 277 1738–1746. 10.1111/j.1742-4658.2010.07597.x 20180803

[B91] Peck S. C. Denger K. Burrichter A. Irwin S. M. Balskus E. P. Schleheck D. (2019). A glycyl radical enzyme enables hydrogen sulfide production by the human intestinal bacterium *Bilophila wadsworthia*. *Proc. Natl. Acad. Sci U.S.A.* 116 3171–3176. 10.1073/pnas.1815661116 30718429 PMC6386719

[B92] Pfenninger-Li X. D. Dimroth P. (1992). NADH formation by Na^+^-coupled reversed electron transfer in *Klebsiella pneumoniae*. *Mol. Microbiol.* 6 1943–1948. 10.1111/j.1365-2958.1992.tb01367.x 1508043

[B93] Ramezani M. Resmer K. L. White R. L. (2011). Glutamate racemization and catabolism in *Fusobacterium varium*. *FEBS J.* 278 2540–2551. 10.1111/j.1742-4658.2011.08179.x 21575137

[B94] Rétey J. Umani-Ronchi A. Seibl J. Arigoni D. (1966). Zum mechanismus der propandioldehydrase-reaktion. *Experientia* 22 502–503. 10.1007/bf01898652 5968618

[B95] Roberts D. L. Frerman F. E. Kim J. J. (1996). Three-dimensional structure of human electron transfer flavoprotein to 2.1-Å resolution. *Proc. Natl. Acad. Sci*. *U.S.A.* 93 14355–14360. 10.1073/pnas.93.25.14355 8962055 PMC26136

[B96] Ruff J. Denger K. Cook A. M. (2003). Sulphoacetaldehyde acetyltransferase yields acetyl phosphate: purification from *Alcaligenes defragrans* and gene clusters in taurine degradation. *Biochem. J.* 369 275–285. 10.1042/bj20021455 12358600 PMC1223080

[B97] Ruzicka F. J. Lieder K. W. Frey P. A. (2000). Lysine 2,3-aminomutase from *Clostridium subterminale* SB4: mass spectral characterization of cyanogen bromide-treated peptides and cloning, sequencing, and expression of the gene kamA in *Escherichia coli*. *J. Bacteriol.* 182 469–476. 10.1128/jb.182.2.469-476.2000 10629195 PMC94298

[B98] Schink B. (1982). *Propionigenium modestum* gen. nov. sp. nov. a new strictly anaerobic, nonsporing bacterium growing on succinate. *Arch. Microbiol.* 133 209–216. 10.1007/bf00415003

[B99] Schmehl M. Jahn A. Meyer zu Vilsendorf A. Hennecke S. Masepohl B. Schuppler M. (1993). Identification of a new class of nitrogen fixation genes in *Rhodobacter capsulatus*: a putative membrane complex involved in electron transport to nitrogenase. *Mol. Gen. Genet.* 241 602–615. 10.1007/bf00279903 8264535

[B100] Schwarz E. Oesterhelt D. Reinke H. Beyreuther K. Dimroth P. (1988). The sodium ion translocating oxalacetate decarboxylase of *Klebsiella pneumoniae*. sequence of the biotin-containing alpha-subunit and relationship to other biotin-containing enzymes. *J. Biol. Chem.* 263 9640–9645. 10.1016/s0021-9258(19)81564-8 2454915

[B101] Schweiger G. Buckel W. (1984). On the dehydration of (*R*)-lactate in the fermentation of alanine to propionate by *Clostridium propionicum*. *FEBS Lett.* 171 79–84. 10.1016/0014-5793(84)80463-9 6586495

[B102] Seedorf H. Fricke W. F. Veith B. Brüggemann H. Liesegang H. Strittmatter A. (2008). The genome of *Clostridium kluyveri*, a strict anaerobe with unique metabolic features. *Proc. Natl. Acad. Sci. U.S.A.* 105 2128–2133. 10.1073/pnas.0711093105 18218779 PMC2542871

[B103] Shibata N. Tamagaki H. Hieda N. Akita K. Komori H. Shomura Y. (2010). Crystal structures of ethanolamine ammonia-lyase complexed with coenzyme B_12_ analogs and substrates. *J. Biol. Chem.* 285 26484–26493. 10.1074/jbc.m110.125112 20519496 PMC2924083

[B104] Shisler K. A. Broderick J. B. (2014). Glycyl radical activating enzymes: structure, mechanism, and substrate interactions. *Arch. Biochem. Biophys.* 546 64–71. 10.1016/j.abb.2014.01.020 24486374 PMC4083501

[B105] Sonnenburg J. Sonnenburg E. (2015). *The Good Gut.* London: Bantam Press.

[B106] Steuber J. Halang P. Vorburger T. Steffen W. Vohl G. Fritz G. (2014a). Central role of the Na(+)-translocating NADH:quinone oxidoreductase (Na(+)-NQR) in sodium bioenergetics of *Vibrio cholerae*. *Biol. Chem.* 295 1389–1399. 10.1515/hsz-2014-0204 25205724

[B107] Steuber J. Vohl G. Casutt M. S. Vorburger T. Diederichs K. Fritz G. (2014b). Structure of the *V. cholerae* Na^+^-pumping NADH:quinone oxidoreductase. *Nature* 516 62–67. 10.1038/nature14003 25471880

[B108] Stickland L. H. (1935). Studies in the metabolism of the strict anaerobes (Genus *Clostridium*): the reduction of proline by *Cl. sporogenes*. *Biochem. J.* 29 288–290. 10.1042/bj0290288 16745669 PMC1266487

[B109] Sucharitakul J. Buckel W. Chaiyen P. (2021a). Rapid kinetics reveal surprising flavin chemistry in the bifurcating electron transfer flavoprotein from *Acidaminococcus fermentans*. *J. Biol. Chem.* 296:100124. 10.1074/jbc.RA120.016017 33239361 PMC7948398

[B110] Sucharitakul J. Buttranon S. Wongnate T. Chowdhury N. P. Prongjit M. Buckel W. (2021b). Modulations of the reduction potentials of flavin-based electron bifurcation complexes and semiquinone stabilities are key to control directional electron flow. *FEBS J.* 288 1008–1026. 10.1111/febs.15343 32329961

[B111] Thauer R. K. Jungermann K. Rupprecht E. Decker K. (1969). Hydrogen formation from NADH in cell-free extracts of *Clostridium kluyveri*. acetyl coenzyme a requirement and ferredoxin dependence. *FEBS Lett.* 4 108–112. 10.1016/0014-5793(69)80208-5 11947158

[B112] Thauer R. K. Jungermann R. Decker K. (1977). Energy conservation in chemotrophic anaerobic bacteria. *Bacteriol. Rev.* 41 100–180. 10.1128/br.41.1.100-180.1977 860983 PMC413997

[B113] Vigil W. Jr. Niks D. Franz-Badur S. Chowdhury N. Buckel W. Hille R. (2021). Spectral deconvolution of redox species in the crotonyl-CoA-dependent NADH:ferredoxin oxidoreductase from *Megasphaera elsdenii*. a flavin-dependent bifurcating enzyme. *Arch. Biochem. Biophys.* 701:108793. 10.1016/j.abb.2021.108793 33587905 PMC8040930

[B114] Vitt S. Prinz S. Hellwig N. Morgner N. Ermler U. Buckel W. (2020). Molecular and low-resolution structural characterization of the Na^+^-translocating glutaconyl-CoA decarboxylase from *Clostridium symbiosum*. *Front. Microbiol.* 11:48011. 10.3389/fmicb.2020.00480 32300335 PMC7145394

[B115] Vogt M. S. Schühle K. Koelzer S. Peschke P. Chowdhury N. P. Kleinsorge D. (2019). Structural and functional characterization of an electron transfer flavoprotein involved in toluene degradation in strictly anaerobic bacteria. *J. Bacteriol.* 201 e00326. 10.1128/JB.00326-19 31405915 PMC6779460

[B116] von Ballmoos C. Wiedenmann A. Dimroth P. (2009). Essentials for ATP synthesis by F_1_F_0_ ATP synthases. *Annu. Rev. Biochem.* 78 649–672. 10.1146/annurev.biochem.78.081307.104803 19489730

[B117] Wang S. Huang H. Moll J. Thauer R. K. (2010). NADP^+^ reduction with reduced ferredoxin and NADP^+^ reduction with NADH are coupled via an electron-bifurcating enzyme complex in *Clostridium kluyveri*. *J. Bacteriol.* 192 5115–5123. 10.1128/jb.00612-10 20675474 PMC2944534

[B118] Wei Y. Zhang Y. (2021). Glycyl radical enzymes and sulfonate metabolism in the microbiome. *Annu. Rev. Biochem.* 90 817–846. 10.1146/annurev-biochem-080120-024103 33823652

[B119] Xing M. Wei Y. Zhou Y. Zhang J. Lin L. Hu Y. (2019). Radical-mediated C-S bond cleavage in C2 sulfonate degradation by anaerobic bacteria. *Nat. Commun.* 10:1609. 30962433 10.1038/s41467-019-09618-8 PMC6453916

[B120] Xu X. Shi H. Gong X. Chen P. Gao Y. Zhang X. (2020). Structural insights into sodium transport by the oxaloacetate decarboxylase sodium pump. *Elife* 9:e53853. 10.7554/eLife.53853 32459174 PMC7274780

[B121] Yu X. Bresser J. Schall I. Djurdjevic I. Buckel W. Wang X. (2012). Development of a satisfactory and general continuous assay for aminotransferases by coupling with (*R*)-2-hydroxyglutarate dehydrogenase. *Anal. Biochem.* 432 127–131. 10.1016/j.ab.2012.09.009 23000002

[B122] Zagalak B. Frey P. A. Karabatsos G. L. Abeles R. H. (1966). The stereochemistry of the conversion of D and L 1,2-propanediols to propionaldehyde. *J. Biol. Chem.* 241 3028–3035. 10.1016/s0021-9258(18)96492-6 4287906

[B123] Zhang H. Dutton P. L. Moser C. C. (2007). Exposing the complex III Q_*o*_ semiquinone radical. *Biochim. Biophys. Acta* 1767 883–887. 10.1016/j.bbabio.2007.04.004 17560537 PMC3554237

